# Multi-omics profiling reveals an auxin–salicylic acid signaling hub driving flavonoid depletion and accelerated vigor loss in tetraploid Chinese cabbage seeds

**DOI:** 10.3389/fgene.2026.1841953

**Published:** 2026-06-08

**Authors:** Chuan Meng, Xiaodong Liu, Fang Wu, Xiaochao Ma, Xiaoshan Chen, Lei Ma, Mingqiu Wang

**Affiliations:** Institute of Cash Crops, Hebei Academy of Agricultural and Forestry Sciences, Shijiazhuang, China

**Keywords:** Brassica rapa, flavonoid metabolism, hormone signaling, multi-omics, polyploidy, seed vigor, transcription factors

## Abstract

**Background:**

Seed vigor, encompassing rapid uniform germination and robust seedling establishment, is critical for crop yield. While induced tetraploidy confers desirable agronomic traits in Chinese cabbage (*Brassica rapa* ssp. *pekinensis*), tetraploid seeds exhibit accelerated viability loss during storage compared to diploid counterparts, imposing substantial economic constraints. The metabolic and regulatory mechanisms underlying this ploidy-associated vigor penalty remain elusive.

**Results:**

TTC assay revealed that seed viability exhibited the most pronounced decline in the 17-year time gradient. Consequently, we prioritized omics analyses on the 19-year and 23-year seeds. Tetraploid seeds demonstrated markedly accelerated viability decline under identical storage conditions, with 2023-harvested tetraploid lots exhibiting the most extensive metabolic rewiring. Comparative transcriptomics revealed ploidy- and year-specific segregation of genes involved in hormone signaling and carbohydrate metabolism. Notably, tetraploid seeds activated a unique auxin--salicylic acid (SA) signaling axis characterized by upregulation of *GH3.3*, *NPR3*, *TGA4*, and IAA family genes, concomitant with elevated indole-3-acetic acid (IAA) and abscisic acid (ABA) accumulation. Genome-wide transcription factor analysis identified ploidy-specific expression patterns in bHLH, WRKY, and bZIP families, with bHLH genes predominantly enriched in tetraploids and bZIP factors associated with diploid seeds. Metabolomic profiling highlighted energy pathway imbalance, specifically starch/sucrose metabolism and glycerophospholipid dysregulation, as the earliest metabolic predictors of vigor loss in tetraploids.

**Conclusion:**

Our findings redefine ploidy-associated seed vigor deterioration as a predictable, metabolically driven syndrome orchestrated by hormone signaling crosstalk and secondary metabolite depletion. The identified auxin--SA signaling axis and energy-metabolism markers provide molecular targets for marker-assisted breeding of high-vigor tetraploid cultivars.

## Introduction

Seed vigor, including rapid, uniform germination and robust seedling establishment under various field conditions, is becoming an increasingly essential agronomic trait for achieving high yields in crops [1]. In Chinese cabbage (*Brassica rapa* ssp. *pekinensis*), the leading leafy vegetable in East Asia, breeders have widely adopted induced tetraploids for their enlarged organs, enhanced stress tolerance, and premium market value (Cai et al., 2024; Niu et al., 2025). However, Tetraploid seeds routinely exhibit faster vigor loss during storage and more erratic field emergence than their diploid counterparts, resulting in substantial economic losses. The metabolic and regulatory basis of this ploidy-associated vigor penalty remains obscure, impeding the development of high-vigor tetraploid cultivars (Zhou et al., 2025; Rasoul et al., 2025).

Previous ‘omics’ studies in other polyploid crops have provided tantalizing clues. Tetraploid Tartary buckwheat (TB) sprouts showed higher biomass compared to diploid sprouts, though their nutritional quality differences remained unclear initially. Both ploidy levels exhibited dynamic changes in water, pigment, mineral, protein, sugar, cellulose, and phenol content (Liu et al., 2023). Tetraploid mulberry cultivars (‘Cheongil’) accumulated higher levels of primary (amino acids, carbohydrates) and secondary metabolites (carotenoids, anthocyanins) than diploids in edible parts. Principal component analysis clearly separated diploid and tetraploid metabolic profiles (Park et al., 2021). Allotetraploidization had observable effects on the metabolome during germination (4–24 h after imbibition), with more pronounced metabolic changes in embryos than in endosperm. The embryo-endosperm metabolomes differed between diploids and tetraploids (Skalska et al., 2021). Tetraploid rice developed a 70% greater rhizosheath under soil-drying conditions than diploids, mediated by *Pseudomonas* bacteria attracted to higher root flavonoid concentrations. This trait was transferable via microbiota transplantation (Xu et al., 2025). Whole-genome duplication in tetraploids influenced triterpene biosynthetic pathways in ginseng and created metabolic differences in Dianthus ecophysiology (Koo et al., 2023). Natural vs. induced tetraploids showed similar meiotic behaviors (Shi et al., 2023). Collectively, these studies indicate that polyploidization imposes broad, context-dependent changes on primary metabolism, secondary metabolism, and plant–microbe interactions, but whether these insights extend to seed vigor *per se* remains untested.

Whether and how harvest year interacts with ploidy to reprogramme the transcriptome–metabolome landscape of Chinese cabbage seeds is unknown. Likewise, the relative contributions of hormone signaling, energy metabolism, and secondary metabolites to the accelerated deterioration of tetraploid seed lots have not been systematically dissected. Closing these gaps is essential for breeding tetraploid lines that combine desirable organ size with robust seed performance. Here, we integrated untargeted metabolomics with Transcriptomics Across four seed lots representing two ploidy levels and two harvest years. We reveal that 2023- tetraploid seeds undergo the largest metabolic rewiring and activate a unique auxin--salicylic acid signaling response that is absent in diploids. The study identifies energy pathway imbalance as key metabolic drivers of ploidy-dependent vigor loss, providing molecular targets for marker-assisted selection of long-vigor tetraploid cultivars.

## Materials and methods

### Plant materials and growth conditions

Seeds of Chinese cabbage were sown on 4 February 2017, in the greenhouse of the Institute of Economic Crops, Hebei Academy of Agriculture and Forestry Sciences, Shijiazhuang, China (38°03′30″N, 114°26′26″E; elevation ∼80 m). Plants were maintained under controlled environmental conditions with nighttime temperatures of 4 °C–7 °C and daytime temperatures of 15 °C–20 °C. Flowering began in late April, after which plants underwent strict self-pollination to ensure genetic homogeneity. Mature seeds were harvested at the end of May and stored at room temperature (20 °C–25 °C, relative humidity 40%–60%) in sealed containers until subsequent analyses.

To assess temporal stability and reproducibility of the observed phenotypic and molecular patterns, the entire cultivation and seed production protocol was independently replicated in 2019 and 2023 under identical environmental and management conditions. Specifically, seeds were sown on 4 February 2019, and 4 February 2023, respectively, with flowering, self-pollination, harvesting, and storage procedures conducted as described above. All seed lots from the three consecutive production years (2017, 2019, and 2023) were subjected to a comprehensive viability assessment. All samples were collected in triplicate (n = 3 biological replicates per ploidy level per year) to ensure statistical robustness. Based on the TTC viability assay results, which revealed significant differences in viability between diploid and tetraploid seeds across temporal replicates, we subsequently conducted integrated transcriptomic and metabolomic analyses of seeds harvested in 2019 and 2023. Specifically, diploid seeds harvested in 2019 (designated as ZA) and 2023 (designated as ZC), as well as tetraploid seeds harvested in 2019 (designated as DA) and 2023 (designated as DC), were subjected to transcriptome sequencing. The same four seed samples (ZA, ZC, DA, and DC) were also analyzed using an untargeted metabolomics.

### Tetrazolium (TTC) Staining for seed viability assessment

Chinese cabbage seeds were pre-soaked in distilled water at 30 °C for 6 h to initiate imbibition. Subsequently, 50 randomly selected seeds were manually peeled and incubated in 0.5% TTC solution at 35 °C for 1 h in darkness. After incubation, the staining solution was discarded, and the seeds were gently rinsed with distilled water to remove residual dye. Viability was evaluated based on the staining pattern of the embryo: seeds with fully or predominantly red-stained embryos were considered viable; those with unstained embryos were deemed non-viable. Partial staining limited to non-critical regions (e.g., portions of cotyledons) without staining of the radicle tip or plumule was classified as indicating germination failure.

### Transcriptome sequencing, RNA extraction, library preparation, and DEG identification

Total RNA was extracted from Chinese cabbage seeds using a modified cetyltrimethylammonium bromide (CTAB)-based protocol optimized for plant tissues rich in polysaccharides and polyphenols. Briefly, 50–100 mg of seeds were rapidly ground to a fine powder in liquid nitrogen using a pre-chilled mortar and pestle. The powder was immediately transferred to a 1.5 mL RNase-free centrifuge tube containing 1 mL of preheated CTAB extraction buffer (2% CTAB, 100 mM Tris-HCl, pH 8.0, 20 mM EDTA, 1.4 M NaCl, 2% polyvinylpyrrolidone) supplemented with β-mercaptoethanol at a final concentration of 2%. The mixture was vortexed vigorously and incubated at 65 °C for 20 min to ensure complete cell lysis and inactivation of endogenous RNases. Following cooling to room temperature, the lysate was extracted with an equal volume of chloroform: isoamyl alcohol (24:1, v/v), mixed thoroughly, and centrifuged at 12,000 x for 10 min at 4 °C. The upper aqueous phase was sequentially re-extracted with equal volumes of phenol:chloroform (25:24, v/v) and chloroform: isoamyl alcohol (24:1, v/v), each followed by centrifugation under identical conditions to remove proteins, polysaccharides, and secondary metabolites. RNA was precipitated from the final aqueous phase by adding an equal volume of isopropanol, incubating at −20 °C for at least 1 h, and centrifuging at 12,000 x for 10 min at 4 °C. The resulting pellet was washed twice with 1 mL of freshly prepared 75% ethanol, with centrifugation at 8,000 x for 5 min between washes, briefly vacuum-dried for 2–4 min to remove residual ethanol, and dissolved in 50 μL of RNase-free water. The purified RNA was stored at −80 °C until further analysis.

RNA purity and integrity were rigorously assessed before downstream applications. Concentration and purity were determined using a NanoDrop 2000 spectrophotometer (Thermo Fisher Scientific, Waltham, MA, USA). High-quality samples exhibited an A260/A280 ratio of 1.8–2.0 and an A260/A230 ratio of approximately 2.2, indicating minimal protein, phenol, or carbohydrate contamination. RNA integrity was evaluated by 1% agarose gel electrophoresis to confirm the presence of distinct 28S and 18S ribosomal RNA bands with an approximate intensity ratio of 2:1, and further quantified using an Agilent 2100 Bioanalyzer (Agilent Technologies, Santa Clara, CA, USA) with the RNA 6000 Nano Kit. Only samples with an RNA Integrity Number (RIN) ≥ 7.0 were deemed suitable for subsequent library construction.

For transcriptome library preparation, mRNA was enriched from 10 ng to 4 μg of total RNA using oligo (dT) magnetic beads (mRNA Capture Beads), fragmented at 94 °C for 5 min in fragmentation buffer, and purified. First-strand cDNA was synthesized using random hexamer primers and Strand Specificity Reagent, followed by second-strand synthesis with dUTP incorporation to maintain strand specificity. The resulting double-stranded cDNA underwent end repair, 3′-adenylation, and adapter ligation using the Hieff NGS® Ultima Dual-mode mRNA Library Prep Kit (Yeasen Biotechnology, Shanghai, China). Ligation products were purified through two sequential rounds of size selection using Hieff NGS® DNA Selection Beads at 0.6× and 0.8× bead-to-sample ratios, and the final libraries were amplified by PCR for 12 cycles using 2× Super Canace II High-Fidelity Mix. Library quality was assessed on an Agilent 2100 Bioanalyzer using the DNA 1000 assay kit to confirm the expected fragment size distribution before paired-end sequencing. Libraries were sequenced on the Illumina sequencing platform by Genedenovo Biotechnology Co., Ltd. (Guangzhou, China). Raw reads were filtered using Fastp (Chen et al., 2018) to obtain high-quality clean reads, which were subsequently aligned to the Brassica rapa reference genome. Gene-level expression was quantified as fragments per kilobase of exon model per million mapped reads (FPKM) (Mortazavi et al., 2008). Read counts per gene were obtained with HTSeq (v0.10.0), and differentially expressed genes (DEGs) were identified using the DESeq2 package (Anders and Huber, 2010) with the default Wald test.

### Metabolite extraction and LC–MS/MS analysis

Fresh tissue (100 mg) was pulverized in liquid nitrogen, and the powder was immediately suspended in 1 mL ice-cold 80% methanol containing 0.1% formic acid. After 5 min on ice, the extract was centrifuged (15,000 × g, 4 °C, 5 min). The supernatant was diluted with LC-MS-grade water to 53% methanol (v/v), vortexed, and then re-centrifuged (15,000 × g, 4 °C, 10 min). The clarified supernatant was transferred to an LC vial and analyzed by UHPLC-QTOF-MS/MS as described (Want et al., 2006).

Chromatographic separation was performed on a Vanquish UHPLC system (Thermo Fisher, Germany) connected to an Orbitrap Q Exactive HF-X mass spectrometer (Thermo Fisher). Full-scan MS and data-dependent MS^2^ spectra were acquired in positive/negative ion modes. Raw files were imported into Compound Discoverer 3.1 (CD 3.1, Thermo Fisher) for retention-time alignment, peak picking, and area integration. Putative annotation was performed by matching accurate mass (<3 ppm) and MS^2^ fragments to the HMDB and LIPID MAPS databases. Multivariate statistical analyses (PCA and PLS-DA) were executed with metaX. Differential metabolites were retained when variable importance in projection (VIP) > 1 from PLS-DA, fold-change ≥ 2 or ≤ 0.5, and Student’s t-test P < 0.05. Intensities were z-score standardized before generating heat maps with the R package pheatmap.

### Statistical procedures

All physiological and molecular traits were analyzed in SPSS 20.0, and graphs were plotted in Graphpad. One-way ANOVA followed by Duncan’s multiple-range test was applied to assess significance (P < 0.05). KEGG pathway enrichment of DEGs and differential metabolites was performed with a hypergeometric test, and terms with P < 0.05 were considered significant.

### Quantitative real-time PCR analysis

To validate the reliability of the differentially expressed genes (DEGs) identified by RNA-Seq, six genes were randomly selected from the DEG list and subjected to quantitative real-time PCR (qRT-PCR) using the same RNA samples. qRT-PCR was performed on an Applied Biosystems 7500 Fast Real-Time PCR System using TransStart® Top Green qPCR SuperMix (TransGen Biotech, Beijing, China). Each 20 μL reaction mixture contained 10 μL of Top Green qPCR SuperMix, 0.4 μL of each primer (10 μM), and 0.4 μL of passive reference dye. All reactions were performed in triplicate with three biological replicates. The thermal cycling conditions were as follows: initial denaturation at 95 °C for 5 min, followed by 40 cycles of denaturation at 95 °C for 15 s, annealing at 58 °C for 20 s, and extension at 72 °C for 30 s. The relative expression levels of the target genes were calculated using the 2^−ΔΔCT^ method, with the *Actin* gene serving as the internal control. Finally, Pearson correlation analysis was conducted to compare the fold changes obtained from qRT-PCR and RNA-Seq.

## Result

### Tetraploid seeds exhibited markedly diminished vigor relative to their diploid counterparts

**FIGURE 1:**
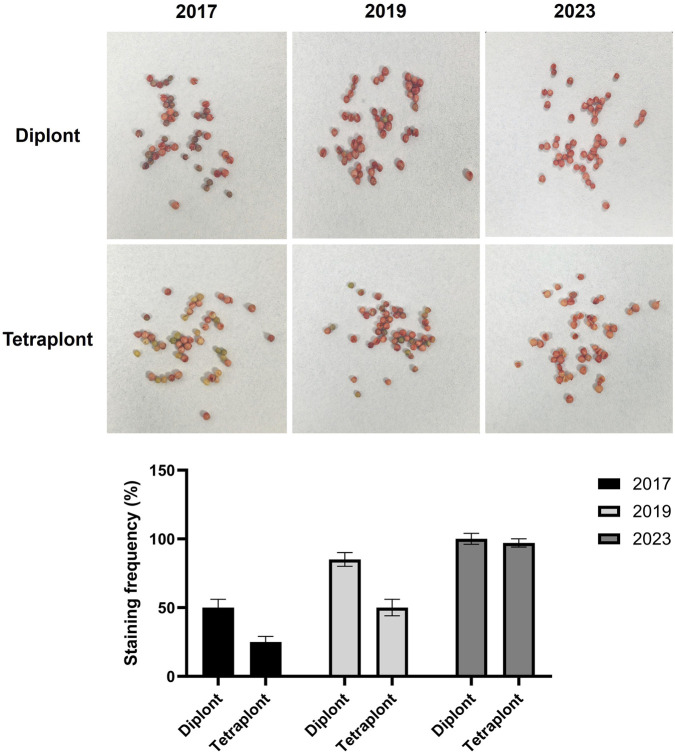
Identification of seed vigor by TTC staining, which includes: Diploid Chinese cabbage seed in 2023, Diploid Chinese cabbage seeds in 2019, Diploid Chinese cabbage seeds in 2017, Tetraploid Chinese cabbage seeds in 2023, Tetraploid Chinese cabbage seeds in 2019 and Tetraploid Chinese cabbage seeds in 2017.

TTC assays revealed that the staining frequency of diploid Chinese cabbage seed lots remained at 100% for the 2023 harvest, declined modestly to 85% for the 2019 lot, and reached approximately 50% for the 2017 lot. In contrast, the most recently harvested tetraploid seed lot (2023) displayed a slightly reduced staining frequency of 97%, already below that of the contemporary diploid lot. This downward trajectory accelerated in older tetraploid lots: the 2019 cohort registered only 50% staining, whereas the 2017 cohort plummeted to 20% (Figure 1). Collectively, these data indicate that tetraploid seeds lose viability more rapidly during storage, implying a shorter shelf-life than diploid seeds.

### Comparative transcriptomics analysis revealed metabolic differences in Chinese cabbage seeds across different years

**FIGURE 2:**
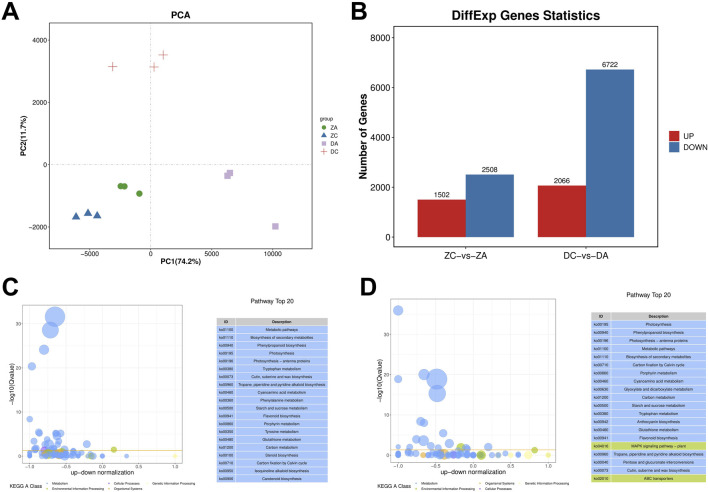
Transcriptome landscape of Chinese cabbage seeds as shaped by ploidy and harvest year. **(A)** Principal-component analysis (PCA) of the four seed lots subjected to transcriptome sequencing. PC1 (74.2%) and PC2 (11.7%) separate samples by ploidy and harvest year. ZA: diploid 2019; ZC: diploid 2023; DA: tetraploid 2019; DC: tetraploid 2023. **(B)** Number of differentially expressed genes (DEGs. **(C)** KEGG functional classification of DEGs in ZC vs. ZA. **(D)** KEGG functional classification of DEGs in DC vs. DA.

To further explore the molecular mechanism underlying reduced seed viability in tetraploid cabbage, we performed transcriptomic analysis. Principal component analysis (PCA) was performed to examine transcriptomic differences among the four seed lots. PC1 explained 74.2% of the total variance, whereas PC2 accounted for 11.7% (Figure 2A). Differential expressed genes analysis revealed a pronounced disparity in Chinese cabbage seeds across different years (Figure 2B; Supplementary Table S1). In the diploid Chinese cabbage seeds, 1502 DEGs were significantly upregulated, and 2508 were downregulated in diploid Chinese cabbage seeds in 2023 (ZC) relative to their counterparts (ZA). A similar trend was observed in tetraploid Chinese cabbage seeds (DC vs. DA), in which tetraploid seeds (DC) in 2023 exhibited 2066 upregulated and 66722 downregulated DEGs compared with tetraploid seeds (DA) in 2019. Furthermore, KEGG enrichment analysis was performed to identify the functions of DEGs. The DEGs in the 2019 and 2023 comparisons (ZC vs. ZA and DC vs. DA) were predominantly mapped to Metabolic pathways, Biosynthesis of secondary metabolites, Phenylpropanoid biosynthesis, etc*.*

### Ploidy-specific expression of bHLH, WRKY, and bZIP transcription factors in Chinese cabbage seeds

**FIGURE 3:**
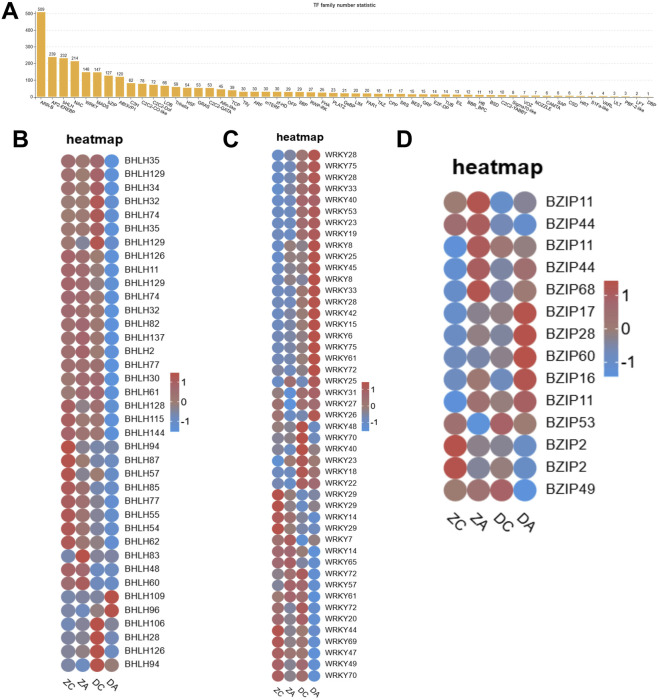
Transcriptional landscape of key TF families. **(A)** TF family number statistics across all differentially expressed genes. **(B–D)** Heatmaps of differentially expressed **(B)** bHLH, **(C)** WRKY, and **(D)** bZIP genes. Heatmap color scale: Z-score standardized expression values (red = high, blue = low). Column order: ZC (diploid 2023), ZA (diploid 2019), DC (tetraploid 2023), DA (tetraploid 2019). Row clustering: Pearson correlation with complete linkage.

Transcription factors (TFs) play pivotal roles in seed development. Here, we systematically categorized TFs (Figure 3A) and conducted in-depth analyses of seed vigor-related genes, with particular emphasis on three major families: bHLH, WRKY, and bZIP. The 38 differentially expressed bHLH genes exhibited complex, sample-specific expression patterns (Figure 3B). A subset of bHLH genes, including bHLH109 and bHLH96, showed pronounced enrichment in the 2019 tetraploid sample (DA), while others were preferentially expressed in diploid seeds (ZA, ZC) or the 2023 tetraploid sample (DC). Notably, eight bHLH members *(BHLH32, BHLH129, BHLH82, BHLH115, BHLH35, BHLH137, BHLH77, and BHLH74*) displayed elevated expression specifically in tetraploid seeds relative to their diploid counterparts, suggesting ploidy-dependent recruitment of this subfamily.

Among the 47 differentially expressed WRKY transcription factors surveyed by the heatmap, the majority exhibited peak transcript abundance in tetraploid Chinese cabbage seeds in 2019 (DA) (Figure 3C), including *WRKY28*, *WRKY75*, *WRKY53*, *WRKY45*, *WRKY8*, *WRKY75*, *WRKY61*, and *WRKY72*. Four WRKY genes, *WRKY48*, *WRKY70*, *WRKY40*, and *WRKY23*, are highly expressed only in tetraploid Chinese cabbage seeds in 2023 (DC).

Fourteen bZIP genes were differentially expressed across the four seed libraries (Figure 3D). Among them, *BZIP11*, *BZIP44*, *BZIP49*, and *BZIP68* accumulated to high levels in both diploid Chinese cabbage seeds (ZA and ZC). Conversely, *BZIP17*, *BZIP28, BZIP16*, and *BZIP60* exhibited pronounced enrichment in the 2019 tetraploid lot (DA), suggesting a stage-specific role for these factors.

### Transcriptome dissection reveals that hormone and sugar genes are segregated by year and ploidy

**FIGURE 4:**
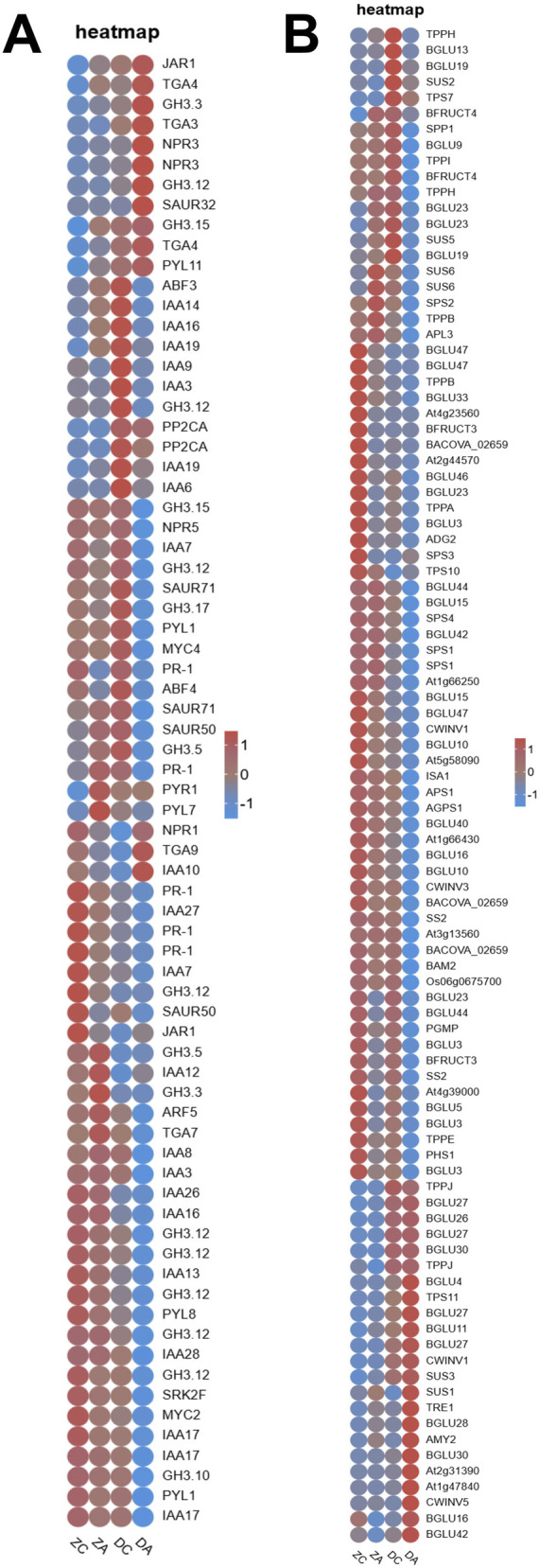
Transcriptional reprogramming of vigor-related pathways. **(A)** Heatmap of plant hormone signal transduction genes (ko04075). **(B)** Heatmap of starch and sucrose metabolism genes (map00500). Heatmap specifications: Z-score standardized expression values (red = high expression, blue = low expression). Column labels (left to right): ZC (diploid 2023), ZA (diploid 2019), DC (tetraploid 2023), DA (tetraploid 2019).

To further dissect the molecular basis of differential vigor between diploid and tetraploid Chinese cabbage seeds, we screened the DEG pool for two pathways known to govern germination: plant-hormone signal transduction and starch/sucrose metabolism. In total, the expression profiles of 148 hormone-related (Figure 4A; Supplementary Table S2) and 96 starch/sucrose-related genes (Figure 4B; Supplementary Table S3) were exhibited across the four seed lots (ZA, ZC, DA, DC). Within the plant hormone module, gene expression exhibited pronounced year- and ploidy-dependent patterns. Specifically, *PR-1*, *IAA27*, *JAR1*, and *SAUR50* showed elevated expression in diploid seeds harvested in 2023 (ZC), whereas *GH3.5*, *IAA12*, *TGA7*, and *GH3.3* were preferentially expressed in the 2019 diploid cohort (ZA). Notably, auxin-responsive genes *IAA14, IAA16, IAA19,* and *IAA3* displayed peak transcript levels in 2023 tetraploid seeds (DC), while salicylic acid-related regulators *TGA3*, *NPR3*, and *GH3*.*12* were enriched in the 2019 tetraploid sample (DA).

In the starch and sucrose metabolism pathway, 2023 tetraploid seeds (DC) exhibited the most robust transcriptional signature associated with cell wall remodeling and sucrose turnover, characterized by high expression of *BGLU19*, *SUS2*, and *BGLU30*. Conversely, the 2023 diploid sample (ZC) showed preferential upregulation of *BGLU33* and *BGLU47*, suggesting divergent metabolic strategies between ploidy levels. An untargeted metabolomic analysis unveils a flavonoid- and lipid-centric metabolite landscape underlying Chinese cabbage seed vigor

**FIGURE 5:**
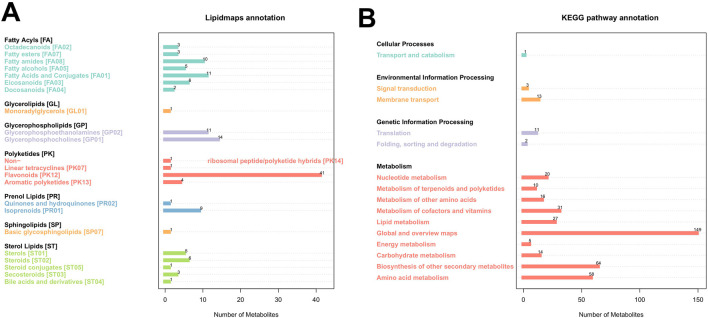
Functional annotation of the untargeted metabolome (1,346 features), including **(A)**. LipidMaps classification and **(B)**. KEGG pathway assignment.

Untargeted metabolomic profiling identified 1346 high-quality features across the four seed lots (Supplementary Table S4). LipidMaps and KEGG annotations were used to analyze the functions of metabolites. LipidMaps annotation analysis indicated 41,14,10 and 11 metabolites were enriched in Flavonoids, Glycerophosphocholines, Fatty amides, and Fatty Acids and Conjugates (Figure 5A). KEGG enrichment analysis revealed that the Global-and-overview maps contained the highest Number of assigned metabolites (149), followed by Biosynthesis of other secondary metabolites (64), amino acid metabolism (58), and metabolism of cofactors and vitamins (31) (Figure 5B).

### Metabolomic profiling reveals enhanced metabolic dysregulation underlying accelerated vigor loss in tetraploid Chinese cabbage seeds

**FIGURE 6:**
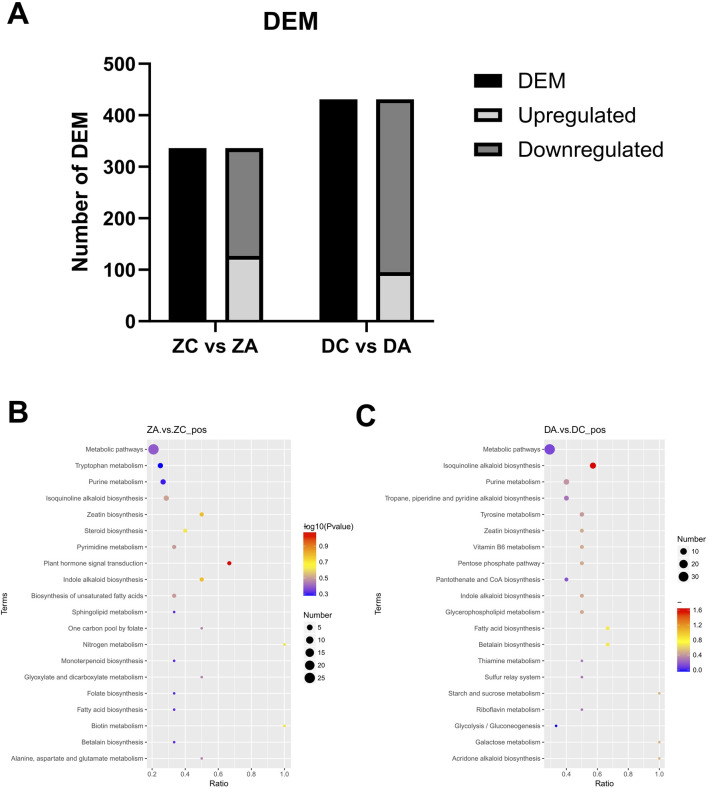
Metabolic rewiring underlying seed vigor divergence. **(A)**. Summary of pairwise comparisons: numbers of differentially expressed metabolites (DEMs) for each contrast. **(B)**. KEGG enrichment analysis of DEGs in ZA vs. ZC. **(C)**. KEGG enrichment analysis of DEGs in DA vs. DC.

To elucidate the metabolic basis for the accelerated vigor loss observed in tetraploid Chinese cabbage seeds, we performed a comprehensive comparative metabolomic analysis (Figure 6A). When comparing the same ploidy level across years, the tetraploid series (DC vs. DA) exhibited a greater metabolic response than the diploid series (ZC vs. ZA). Specifically, DC vs. DA yielded 431 DEMs (95 upregulated and 336 downregulated), whereas ZC vs. ZA yielded 336 DEMs (127 upregulated and 209 downregulated). Furthermore, KEGG enrichment analysis was performed to examine the functions of DEMs. In ZA vs. ZC, DEMs were enriched in tryptophan metabolism, purine metabolism, zeatin biosynthesis, and plant hormone signal transduction (Figure 6B). In DA vs. DC, the most significantly enriched routes were starch and sucrose metabolism, pentose phosphate pathway, glycerophospholipid metabolism, and fatty acid biosynthesis (Figure 6C).

### Multi-omics dissection reveals ploidy- and age-dependent hormonal regulatory architecture governing seed vigor

**FIGURE 7:**
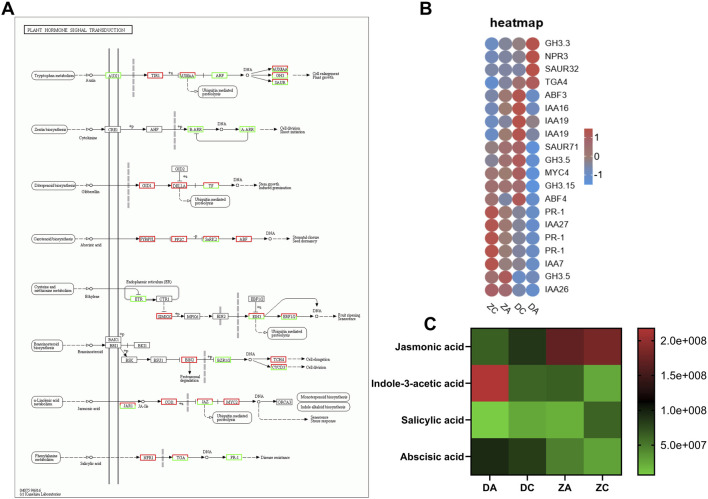
Integrated hormone-centric multi-omics map. **(A)** Plant hormone signal transduction pathway (ko04075) highlighting differentially expressed genes. **(B)** Heatmap of hormone-related differentially expressed genes. **(C)** Heatmap of hormone-related differentially expressed metabolites (IAA, ABA, SA, JA). Heatmap color scales: Z-score standardized values. Gene heatmap **(B)** red = high expression, blue = low expression; columns: ZC, ZA, DC, DA. Metabolite heatmap **(C)** color intensity represents relative abundance; columns: DA, DC, ZA, ZC.

Integrated transcriptomic and metabolomic analyses demonstrated dynamic remodeling of phytohormone signaling networks across developmental stages and ploidy levels. In 2019-harvested tetraploid seeds (DA), Auxin (IAA) signaling components *GH3.3*, *NPR3*, and *TGA4* exhibited significant upregulation compared to diploid counterparts, coinciding with elevated IAA and abscisic acid (ABA) accumulation (Figures 7A–C). Concurrently, *IAA27*, *IAA7*, and pathogenesis-related gene *PR-1* showed distinct expression patterns specific to the tetraploid 2019 cohort. The ABA-responsive transcription factor *ABF3* was differentially enriched in DA samples, indicating enhanced ABA signaling in tetraploid seeds.

Comparative analysis across harvest years revealed that 2023-harvested tetraploid seeds (DC) exhibited peak transcript levels of *IAA14*, *IAA16*, *IAA19*, and *IAA3*. In contrast, salicylic acid (SA)-related regulators *TGA3*, *NPR3*, and *GH3*.*12* were preferentially expressed in the 2019 tetraploid sample (DA). Four WRKY genes (*WRKY48*, *WRKY70*, *WRKY40*, and *WRKY23*) showed exclusive high expression in DC samples.

Metabolomic profiling corroborated these transcriptional patterns, with IAA and ABA levels significantly higher in tetraploid seeds than in diploid controls at equivalent storage time points. The integration of transcriptomic and metabolomic datasets identified a ploidy-specific auxin--SA signaling response with potential JA crosstalk, characterized by coordinated upregulation of hormone biosynthesis and signal transduction genes in tetraploid seeds that was absent in diploid counterparts.

### Verification of sequence data

**FIGURE 8:**
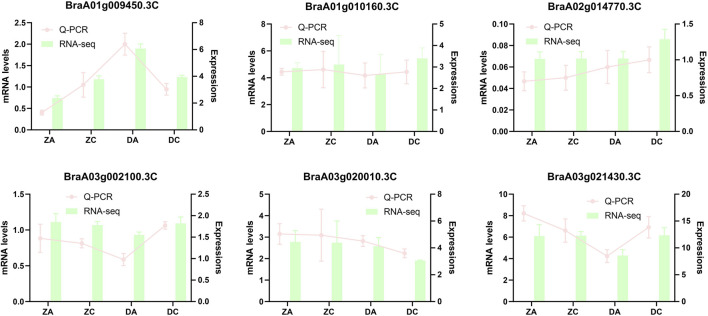
Correlation between sequencing data and quantitative RT-PCR data.

To independently validate the RNA-Seq results, quantitative real-time PCR (qRT-PCR) was performed using the same RNA samples employed for transcriptome sequencing. Six differentially expressed genes (DEGs) were randomly selected across a range of expression patterns for qRT-PCR verification. Gene-specific primers were designed using NCBI Primer-BLAST, and their sequences, together with those of the reference gene, *Actin*, are provided in Supplementary Table S5. The relative expression levels of the six selected DEGs determined by qRT-PCR exhibited a strong positive correlation with the corresponding RNA-Seq data (Figure 8), confirming the high reliability and accuracy of the transcriptome sequencing results.

## Discussion

Seed vigor is a multigenic trait that determines a crop’s commercial value, yet the mechanisms linking ploidy level, storage time, and germination performance remain poorly understood. By integrating transcriptome and untargeted metabolome profiles of diploid and tetraploid Chinese cabbage seed lots spanning three harvest years and two developmental checkpoints, we provide the first comprehensive view of how genome duplication reshapes the molecular trajectory of seed aging. Four central conclusions emerge: (i) tetraploids lose viability faster than diploids under identical storage conditions; (ii) the deterioration is accompanied by a unique, year-specific rewiring of auxin--salicylic acid (SA) signaling, with potential involvement of jasmonic acid (JA) crosstalk; (iii) Starch/sucrose metabolic imbalance are the earliest metabolic predictors of vigour loss; Collectively, these data redefine ploidy-associated vigour penalty as a predictable, metabolically driven syndrome and nominate molecular targets for breeding high-vigour tetraploid cultivars.

The transcriptomic dissection reveals a clear segregation of hormone signaling and sugar metabolism genes by both ploidy level and year in Chinese cabbage seeds, providing molecular insights into the differential germination vigor between diploid and tetraploid varieties. The Identification of 148 hormone-related and 96 carbohydrate-related DEGs demonstrates the coordinated regulation of these two fundamental pathways in seed germination physiology [17]. Conversely, the peak expression of negative regulators *RGL2* (GA signaling repressor) and *NPR1* (SA signaling node) in 2019 tetraploid dry seeds (DA) indicates potential suppression of germination signals in these samples (Han et al., 2025). The differential expression of core ABA/JA signaling components (*SRK2C*, *ASK7*) across seed lots further highlights the temporal modulation of hormone crosstalk during germination (Liu et al., 2025).

In the starch/sucrose metabolism pathway, the distinct transcriptional signatures between ploidy levels reveal fundamental differences in energy mobilization strategies. The strong cell-wall remodeling and sucrose turnover signature in 2023 tetraploid seeds (DC), characterized by high *BGLU19*, *SUS2*, and *BGLU30* expression, suggests an enhanced capacity for cell wall modification and sucrose conversion (Yu et al., 2025). The diploid-specific upregulation of *BGLU33* and *BGLU47* in ZC samples indicates that alternative β-glucosidase isoforms may be preferentially employed in diploid seed germination (Song et al., 2025). The year-dependent expression patterns suggest environmental factors may interact with ploidy to shape the germination transcriptome. The 2023 samples generally showed greater expression of growth-promoting genes than the 2019 samples, potentially reflecting differences in maternal environment or seed storage conditions (Wang et al., 2022). This temporal variation underscores the importance of considering both genetic (ploidy) and environmental factors in seed vigor studies. These findings align with emerging models of seed germination as a hormone-sugar crosstalk process, in which auxin-GA-ABA balance coordinates with starch/sucrose metabolism to regulate germination efficiency. The ploidy-specific expression patterns suggest that tetraploids may achieve higher vigor through enhanced auxin signaling, coupled with more efficient cell wall remodeling and sugar-conversion capacity (Yu et al., 2025). Future functional studies targeting the key identified genes (e.g., TIFY family members, BGLU isoforms) could further validate their roles in ploidy-dependent vigor regulation.

The differential expression patterns of bHLH, WRKY, and bZIP transcription factors (TFs) between diploid and tetraploid Chinese cabbage seeds reveal intriguing ploidy-specific regulatory mechanisms governing seed germination and dormancy. This ploidy-dependent partitioning of TF networks aligns with emerging evidence that transcription factor families often function in combinatorial networks to regulate complex developmental processes (Xing et al., 2025). Notably, eight bHLH members *(BHLH32, BHLH129, BHLH82, BHLH115, BHLH35, BHLH137, BHLH77, and BHLH74*) were preferentially expressed in tetraploid relative to diploid seeds, suggesting ploidy-dependent recruitment of this subfamily. This selective enrichment, rather than global bHLH upregulation, indicates that specific bHLH paralogs may have been co-opted during tetraploidization to regulate vigor-related processes. This finding corroborates previous studies that have identified bHLH factors as key regulators of various developmental processes, including flavonoid biosynthesis and stress responses (Imran et al., 2022). The preferential expression of these bHLH members in tetraploids may indicate their role in overcoming germination-inhibition mechanisms that remain active in diploid seeds. The WRKY family exhibited particularly striking ploidy-specific partitioning, with *WRKY28*, *WRKY33*, *WRKY15*, *WRKY75*, and *WRKY31* showing tetraploid-specific expression patterns. This aligns with established roles of WRKY factors in stress responses and developmental transitions. The contrasting expression of other WRKY members (*WRKY29*, *WRKY44*, *WRKY72*, *WRKY57*, *WRKY48*, *WRKY20*, and *WRKY27*) in diploid seeds suggests that these may function in maintaining dormancy, consistent with reports of WRKY proteins participating in complex regulatory networks with other TF families (Wang et al., 2021). The bZIP results reveal particularly interesting dynamics, with *BZIP11*, *BZIP44*, *BZIP49*, and *BZIP68* showing strong association with diploid seeds. This supports the emerging paradigm of bZIP factors as central regulators of seed dormancy programs (Khan et al., 2022). The stage-specific enrichment of *BZIP11*, *BZIP28*, and *BZIP60* in tetraploid seeds (DA) suggests that these factors may participate in preparatory processes preceding germination, consistent with findings that bZIP11 regulates early seed development in Brassica species (Khan et al., 2022). The observed ploidy-dependent TF expression patterns may reflect evolutionary adaptations in gene regulatory networks. Tetraploids appear to have developed distinct transcriptional programs favoring germination, while diploids maintain expression profiles conducive to dormancy. This aligns with studies showing that polyploidization often leads to the rewiring of transcriptional networks (Ziegler et al., 2025). The specific recruitment of bHLH and WRKY factors in tetraploids, versus bZIP retention in diploids, suggests that these TF families may represent key nodes in ploidy-specific regulatory circuits controlling germination competence. These findings contribute to our understanding of how transcription factor networks evolve in polyploids and how they may influence important agronomic traits, such as germination vigor. The identified TF candidates provide valuable targets for future functional studies aimed at manipulating seed dormancy and germination characteristics in Brassica crops.

Our multi-omics dissection uncovered a sophisticated, ploidy- and age-dependent hormonal regulatory architecture that underpins seed vigor in Chinese cabbage. Integrated transcriptomic and metabolomic analyses highlighted dynamic remodeling of phytohormone signaling networks across developmental stages and environmental contexts. Notably, Auxin (IAA) signaling components such as *GH3.3*, *NPR3*, and *TGA4* were upregulated in 2019 tetraploid seeds (DA), coinciding with elevated IAA and abscisic acid (ABA) accumulation—hormones known to modulate seed dormancy and stress adaptation (Mei et al., 2023), alongside distinct expression of *IAA27*, *IAA7*, and *PR-1*. The differential enrichment of ABF3—a key ABA-responsive transcription factor—in DA further suggests enhanced ABA signaling in tetraploids, potentially contributing to improved desiccation tolerance or longevity (Wang et al., 2024). These findings align with the established roles of JA and SA in mediating biotic and abiotic stress responses (Shikha et al., 2023). At the same time, the ploidy-specific hormone profiles imply that polyploidization rewires hormonal crosstalk to optimize seed performance under varying storage or environmental conditions. Thus, the interplay between ploidy level, harvest year, and phytohormone dynamics orchestrates a nuanced regulatory landscape governing seed vigor.

Our multi-omics dissection reveals a highly coordinated, ploidy- and age-dependent hormonal regulatory architecture that critically governs seed vigor in Chinese cabbage. The integration of transcriptomic and metabolomic data demonstrates that polyploidization induces profound rewiring of phytohormone signaling networks, particularly involving Auxin (IAA) and abscisic acid (ABA), which are central to seed dormancy, stress adaptation, and longevity (Gong et al., 2024). The upregulation of IAA-responsive genes (e.g., *GH3.3*, *IAA27*, *IAA7*) and ABA-associated factors (e.g., *ABF3*, *NPR3*, *TGA4*) in tetraploid seeds from 2019 (DA) correlates with elevated IAA and ABA levels, suggesting a synergistic hormonal milieu that enhances seed resilience during storage or under stress (Chen et al., 2024). This is further supported by the enrichment of pathogenesis-related *PR-1*, implicating salicylic acid (SA) signaling in vigor maintenance—a pathway known to intersect with both ABA and jasmonate (JA) responses in mediating biotic and abiotic stress tolerance (Zhang et al., 2025). Critically, the observed differences between harvest years and ploidy levels underscore that seed vigor is not governed by static genetic programs but by dynamic, context-dependent hormonal crosstalk shaped by both genomic dosage and environmental history. These findings position polyploidy as a key modulator of hormonal network plasticity, offering a mechanistic basis for improved seed performance in polyploid crops and highlighting potential targets for breeding high-vigor varieties resilient to aging and climate variability.

Mechanistic insights into the auxin-SA signaling axis and associated metabolic reprogramming. The ploidy-specific transcriptional response in tetraploid seeds potentially regulates seed vigor through three interconnected routes: (i) ABA signaling reinforcement—elevated IAA and ABA accumulation, together with upregulation of ABF3, suggests auxin-ABA synergism reinforcing dormancy programs; (ii) ROS homeostasis modulation—SA pathway activation (*NPR3, TGA4*) may redirect ROS signaling to stress-responsive pathways, pending experimental validation; (iii) Carbohydrate allocation reprogramming—IAA-responsive genes (*IAA14, IAA16, IAA19*) may antagonize GA-driven starch mobilization, aligning with observed starch/sucrose dysregulation. While JA pathway genes (*LOX, AOS, MYC2*) were not differentially expressed, post-transcriptional involvement cannot be excluded. Metabolomic data identified glycerophospholipid and starch/sucrose pathway dysregulation as the primary metabolic features of tetraploid deterioration; however, these associations are correlative, as physiological validation (membrane permeability, respiratory rate) was not performed. The causal hierarchy between hormone signaling and metabolic imbalance remains to be established through targeted experiments.

## Conclusion

By integrating transcriptome and untargeted metabolome data across two harvest years, we demonstrate that tetraploid Chinese cabbage seeds lose vigor faster than diploids, primarily driven by a unique auxin--salicylic acid signaling axis with associated hormone crosstalk, and energy pathway imbalance. These findings redefine ploidy-associated vigor loss as a predictable metabolic syndrome and provide hormone and TF markers for breeding high-vigor tetraploid cultivars.

## Data Availability

The original contributions presented in the study are publicly available. This data can be found in the NCBI SRA repository with the accession number PRJNA1117348.
